# State-level trends in sudden unexpected infant death and immunization in the United States: an ecological study

**DOI:** 10.1186/s12887-021-02733-w

**Published:** 2021-06-11

**Authors:** Jacqueline Müller-Nordhorn, Konrad Neumann, Thomas Keil, Stefan N. Willich, Sylvia Binting

**Affiliations:** 1 Institute for Social Medicine, Epidemiology and Health Economics, Charité - Universitätsmedizin Berlin, Charitéplatz 1, 10117 Berlin, Germany; 2 Bavarian Cancer Registry, Bavarian Health and Food Safety Authority, Nuremberg, Germany; 3 Institute of Biometry and Clinical Epidemiology, Charité - Universitätsmedizin Berlin, Berlin, Germany; 4 Institute for Clinical Epidemiology and Biometry, University of Wuerzburg, Wuerzburg, Germany; 5 Institute for Health Resort Medicine and Health Promotion, Bavarian Health and Food Safety Authority, Bad Kissingen, Germany

**Keywords:** Sudden unexpected infant death, Vaccination coverage, Time trends

## Abstract

**Background:**

Sudden unexpected infant death (SUID) continues to be a major contributor to infant mortality in the United States. The objective was to analyze time trends in SUID and their association with immunization coverage.

**Methods:**

The number of deaths and live births per year and per state (1992–2015) was obtained from the Centers for Disease Control and Prevention (CDC). We calculated infant mortality rates (i.e., deaths below one year of age) per 1000 live births for SUID. We obtained data on immunization in children aged 19–35 months with three doses or more of diphtheria-tetanus-pertussis (3+ DTP), polio (3+ Polio), and *Haemophilus influenzae* type b (3+ Hib) as well as four doses or more of DTP (4+ DTP) from the National Immunization Survey, and data on infant sleep position from the Pregnancy Risk Assessment Monitoring System (PRAMS) Study. Data on poverty and race were derived from the Current Population and American Community Surveys of the U.S. Census Bureau. We calculated mean SUID mortality rates with 95% confidence interval (CI) as well as the annual percentage change using breakpoint analysis. We used Poisson regression with random effects to examine the dependence of SUID rates on immunization coverage, adjusting for sleep position and poverty (1996–2015). In a second model, we additionally adjusted for race (2000–2015).

**Results:**

Overall, SUID mortality decreased in the United States. The mean annual percent change was − 9.6 (95% CI = − 10.5, − 8.6) between 1992 and 1996, and − 0.3 (95% CI = − 0.4, − 0.1) from 1996 onwards. The adjusted rate ratios for SUID mortality were 0.91 (95% CI = 0.80, 1.03) per 10% increase for 3+ DTP, 0.88 (95% CI = 0.83, 0.95) for 4+ DTP, 1.00 (95% CI = 0.90, 1.10) for 3+ polio, and 0.95 (95% CI = 0.89, 1.02) for 3+ Hib. After additionally adjusting for race, the rate ratios were 0.76 (95% CI = 0.67, 0.85) for 3+ DTP, 0.83 (95% CI = 0.78, 0.89) for 4+ DTP, 0.81 (95% CI = 0.73, 0.90) for 3+ polio, and 0.94 (95% CI = 0.88, 1.00) for 3+ Hib.

**Conclusions:**

SUID mortality is decreasing, and inversely related to immunization coverage. However, since 1996, the decline has slowed down.

## Background

Approximately 3600 infants die from sudden unexpected infant death (SUID) in the United States per year [1, 2]. The decrease in SUID observed during the 1990s has slowed down, with a large variation in regional trends between states and even an increase observed in some states [3]. SUID includes sudden infant death syndrome (SIDS), accidental suffocation and strangulation in bed, and other ill-defined causes of mortality [2]. While SIDS mortality continues to decrease - albeit slowly -, mortality from accidental suffocation and strangulation in bed and other ill-defined causes has increased in recent years [2–4].

The large decline in mortality during the 1990s - in particular with regard to SIDS mortality - has been attributed to the “Back to Sleep” campaigns [2, 5, 6]. They promoted a change from the prone or side sleep position in infants to the supine sleep position [7, 8]. About 70% of infants are now sleeping in the supine position [9]. A non-supine sleep position is, however, considered only an extrinsic risk factor, but not the cause, of SIDS [10]. The cause of SIDS is still unknown. In addition to the non-supine sleep position and other sleep-related factors such as soft bedding, bed sharing, or overheating, potentially modifiable risk factors are parental smoking, lack of pacifier use, and lack of immunizations [2, 10–12]. The risk of SIDS is higher in Black and Native American infants as well as in populations suffering from socioeconomic deprivation [2].

Meta-analyses of case-control and cohort studies have shown a reduced risk of SIDS associated with diphtheria-tetanus-pertussis (DTP), polio, and *Haemophilus influenzae* type b (Hib) immunization [11, 12]. Compared to the direct protective effect of immunization in individuals, less evidence is available with regard to the indirect protective effect. One ecological study has shown a reduced risk of SIDS mortality associated with higher population coverage in the United States [6]. However, multiple data sources for immunization coverage had to be used. Since the mid-1990s, the National Immunization Survey (NIS) has assessed immunization coverage in the United States at the state-level in a standardized way [13]. The association between SUID mortality at the state-level and population coverage with the DTP, polio, and Hib vaccines is not clear. The objective of the present study was to analyze the association between SUID and immunization coverage at the state-level, adjusting for sleep position, poverty, and race.

## Methods

### Study design

We compared trends in mortality rates from SUID as well as SIDS, accidental suffocation and strangulation in bed, and other ill-defined and unspecified causes of mortality at the state-level in the United States. The time period of the analysis was 1992–2015. We accessed the number of infant deaths in the respective diagnostic codes as well as the number of live births from the Centers for Disease Control and Prevention (CDC) Wide-ranging Online Data for Epidemiologic Research (WONDER) website [1]. Infant deaths were defined as deaths among children below one year of age. The following International Classification of Deaths (ICD) codes were used: the ICD-10 codes R95 (SIDS), W75 (accidental suffocation and strangulation in bed), and R99 (other ill-defined and unspecified causes of mortality) for the years 1999–2015, and the respective ICD-9 codes 798.0, E913.0, and 799.9 for the years 1992–1998 [1, 4].

### Collection of data

#### Vaccination coverage

The CDC started the National Immunization Survey (NIS) in April 1994 [13]. The NIS collects data from the 50 states and the District of Columbia quarterly. Data at the state-level are available from 1994 onwards [13, 14]. Yearly coverage levels are published on the CDC website as public use files [14]. The NIS is a random-digit-dialing telephone survey including households with children aged 19–35 months. The data are validated with the immunization history of the child, which is obtained from the family’s health care provider. Adjustments are made for non-response and for the exclusion of households without a telephone [13].

We included immunization coverage with the DTP, polio, and Hib vaccines. During the first six months of life, three doses of DTP vaccine (month 2, 4, 6), three doses of poliovirus vaccine (month 2, 4, 6–18), and three doses of Hib vaccine (month 2, 4, 6 and/or 12–15, depending on the product type) are recommended [15]. Data was obtained from the NIS on three doses or more of DTP (3+ DTP), polio (3+ Polio), and Hib (3+ Hib). We also included immunization coverage with the 4th dose of DTP (month 15–18, 4+ DTP) as an additional marker of indirect protection. Over time, recommended vaccine schedules changed. DTaP with an acellular pertussis antigen became the preferred vaccine formulation and replaced the DTP vaccine in 1999 [16]. Inactivated poliovirus vaccine was recommended instead of oral poliovirus from 1999/2000 onwards [16, 17].

#### Infant sleep position

The Pregnancy Risk Assessment Monitoring System (PRAMS) is an on-going state-based surveillance system of maternal behaviors, attitudes, and experiences [18]. The CDC’s Division of Reproductive Health conducts PRAMS in collaboration with state health departments. PRAMS is a mixed-mode survey using both mail and telephone data collection. Based on birth certificate records, representative samples of all women who delivered a live-born infant are drawn, stratified for maternal age, race/ethnicity, and infant birth weight. Annual sample sizes range from approximately 1000 to 3000 women per state. Statistical weighting schemes allow estimates from these groups to be combined to obtain state-level estimates. In PRAMS, the weighted response rates ranged from 47 to 74%, with a median of 61% (2014) [18].

Data on the percentage of children sleeping in the supine position was available in PRAMS at the state-level for the years 1996–2015 [19]. We combined data on sleep position from New York State and New York City. We imputed missing values using multiple imputation with m = 5 imputation samples. Results from the m = 5 separate analyses on the imputation samples were pooled by Rubin’s rule [20]. We excluded states without at least a single value on sleep position. For the states Arizona, California, Connecticut, District of Columbia, Idaho, Indiana, Kansas, Kentucky and Nevada, no data on sleep position were available (22.3% of the live births).

#### Poverty and race

Poverty (%) per year and by state were based on historical data from the Annual Social and Economic Supplements of the Current Population Survey of the U.S. Census Bureau [21]. Poverty was defined as a family income below a certain threshold. The Census Bureau used a set of income thresholds taking into account the size of the family and the age of its members and compared the family’s money income before taxes to the respective threshold to determine poverty [22].

We obtained data on race from historical surveys of the American Community Survey of the U.S. Census Bureau [23]. Data at the state-level was available for the years 2000 to 2015 [24, 25]. The Census Bureau used the classifications according to the 1997 Office of Management and Budget standards on race and ethnicity [23]. The written responses to the question on race were classified as follows: White, Black or African American, American Indian or Alaska Native, Asian, as well as Native Hawaiian or Other Pacific Islander. The reporting is based upon self-identification.

### Statistical analyses

We calculated mortality rates for SUID dividing the number of deaths by the number of live births (1992–2015). For the description of time trends, we aggregated the data at the state-level into U.S. census divisions (New England, Middle Atlantic, East North Central, West North Central, South Atlantic, East South Central, West South Central, Mountain, Pacific) [26]**.** We performed breakpoint analysis for simple Poisson regression. A breakpoint divides the period under consideration into two intervals with different annual percentage change in mortality rates. We calculated the position of the breakpoint and the annual percentage change before and after the breakpoint using an iterative algorithm, implemented in the R package “segmented” [27]**.** Furthermore, the breakpoint analysis provided estimates of the mortality rates for SUID in 1992, at the year of the breakpoint, and in 2015 with 95% confidence intervals. We provided time trends both on the national level as well as on the level of the nine divisions.

We used Poisson regression with random effects at the state-level to examine the dependence of SUID rates on the covariates immunization coverage, infant sleep position, poverty level, and race. Each covariate was first tested with a simple Poisson model, i.e., a model with intercepts (random and fixed) and one independent variable. We then calculated multiple Poisson regression models, adding the potential confounders sleep position and poverty as independent variables (Model 1, years 1996–2015). For the years 2000–2015, we additionally adjusted for the variable race (Model 2). In simple regression analyses, we included the main groups of White, Black and Asian Americans as independent variables. We excluded the groups American Indians or Alaska Natives as well as Native Hawaiians or Other Pacific Islanders since percentages were too small in most states. However, in multiple regression analyses, we used the first two principal components (PC1 and PC2), calculated from all five groups describing the racial composition of the population. The random intercepts for the years 1996 to 2015 were included in the model equation to account for unknown confounders correlated with time. We calculated exp.(10*β), the rate ratio per 10% change in the independent variable, and the respective two-sided 95% confidence interval for each independent variable. The rate ratio was the relative change in SUID and SIDS deaths for each 10% increase of the independent variable. Since the number of live births differed significantly between larger and smaller US states, the likelihood-based calculation of two-sided 95% confidence intervals implemented in the R function glmmPQL seemed to inflate α-levels. Therefore, we replaced it with a more robust resampling (bootstrap) method, resulting in confidence intervals that were more conservative. We excluded states without any information on sleep position from all analyses in order to use a uniform data basis. As sensitivity analyses, we performed the regression analyses with SIDS as dependent variable - to determine the robustness of the results - as well as unadjusted simple regression analyses including all states.

We used the statistical software R version 3.4.1 for all analyses. For simple and multiple regression analyses, we used the R function glmmPQL from the R package “MASS”, and for the breakpoint analysis the package “segmented”. The two-sided confidence level was set at 95%. We did not adjust for multiple testing due to the exploratory nature of the study.

## Results

**Fig. 1:**
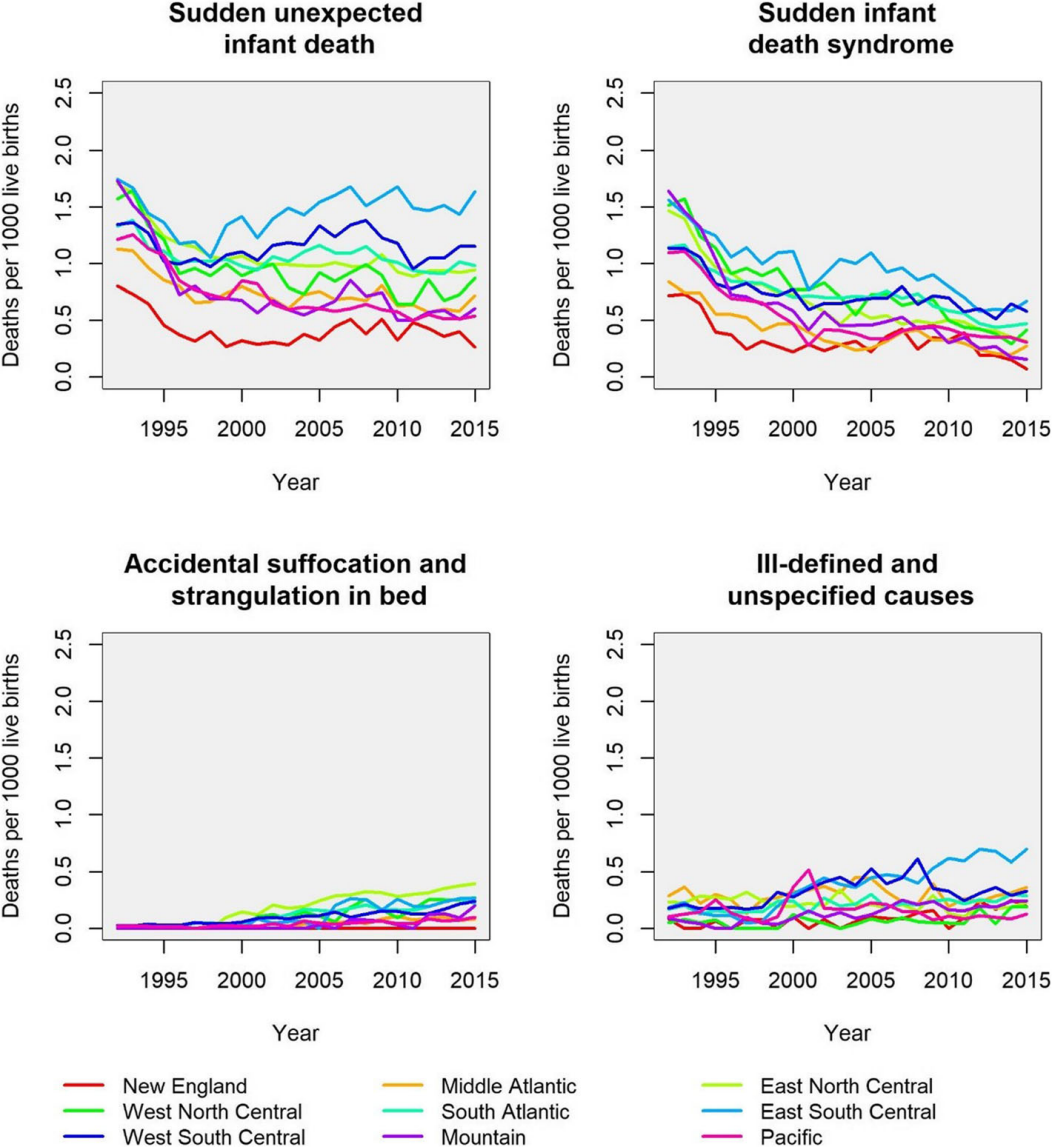
Mortality rates from sudden unexpected infant death and its components over time, according to U.S. census divisions [1, 26]. Excluding the states Arizona, California, Connecticut, District of Columbia, Idaho, Indiana, Kansas, Kentucky and Nevada from the analyses

Between 1992 and 2015, overall mortality rates from SUID decreased from 1.47 / 1000 live births (95% confidence interval [CI] = 1.44, 1.51) to 0.91 (95% CI = 0.90, 0.93) in the United States (Table 1). The annual percentage decrease was − 9.6 (95% CI = − 10.5, − 8.6) between 1992 and 1996, slowing down to − 0.3 (95% CI = − 0.4, − 0.1) from 1996 onwards. Trends in SUID mortality differed among divisions (Table 1). Whereas SUID rates continued to decrease or stagnated in six out of the nine divisions since the mid-1990s, rates increased slightly in three divisions (New England, East South Central, West South Central) after the respective year of the breakpoint. Figure 1 shows regional trends in mortality rates separately for the diagnoses SIDS, accidental suffocation and strangulation in bed, and other ill-defined or unspecified cause of mortality.

**Table 1:** Breakpoint Analyses of Mortality Rates from Sudden Unexpected Infant Death (SUID) in U.S. Census Divisions, 1992–2015^1^

	SUID / 1000 live births (95% CI^b^)				Before breakpoint	After breakpoint
	Year					
Census divisions^a^	1992	Year of breakpoint		2015	Annual % change (95% CI)	Annual % change (95% CI)
New England	0.84 (0.73, 0.97)	1997	0.24 (0.21, 0.28)	0.46 (0.40, 0.53)	−20.4 (− 25.0, − 15.4)	3.7 (2.2 to 5.1)
Middle Atlantic	1.16 (1.09, 1.23)	1997	0.73 (0.69, 0.77)	0.64 (0.61, 0.68)	−9.0 (−11.4, −6.4)	−0.7 (− 1.1, − 0.2)
East North Central	1.74 (1.65, 1.83)	1997	1.11 (1.06, 1.17)	0.92 (0.89, 0.96)	− 9.3 (− 11.3, − 7.3)	−1.0 (− 1.4, − 0.6)
West North Central	1.74 (1.61, 1.89)	1996	0.94 (0.87, 1.02)	0.72 (0.68, 0.78)	−13.2 (− 16.3, − 9.9)	−1.4 (− 2.0, − 0.8)
South Atlantic	1.34 (1.27, 1.42)	1996	1.05 (1.02, 1.11)	1.01 (0.99, 1.07)	−6.3 (− 9.2, − 3.3)	− 0.2 (− 0.5, 0.1)
East South Central	1.78 (1.63, 1.94)	1996	1.31 (1.24, 1.41)	1.68 (1.60, 1.81)	−7.1 (− 10.6, − 3.5)	1.3 (0.8, 1.9)
West South Central	1.41 (1.32, 1.50)	1995	1.09 (1.05, 1.15)	1.19 (1.15, 1.25)	−8.2 (− 11.5, − 4.7)	0.4 (0.1, 0.8)
Mountain	1.96 (1.77, 2.16)	1996	0.77 (0.70, 0.87)	0.46 (0.42, 0.51)	−20.4 (− 24.1, − 16.5)	−2.7 (− 3.5, − 1.9)
Pacific	1.80 (1.65, 1.96)	1999	0.83 (0.75, 0.93)	0.66 (0.60, 0.72)	−11.2 (− 13.6, − 8.8)	−1.4 (− 2.3, − 0.5)
USA total	1.47 (1.44, 1.51)	1996	0.97 (0.95, 0.98)	0.91 (0.90, 0.93)	−9.6 (− 10.5, − 8.6)	− 0.3 (− 0.4, − 0.1)

^a^ Excluding the states Arizona, California, Connecticut, District of Columbia, Idaho, Indiana, Kansas, Kentucky and Nevada from the analyses

^b^ Confidence intervals (CI) were calculated using parametric bootstraps with *N* = 1000,000 runs

**Fig. 2:**
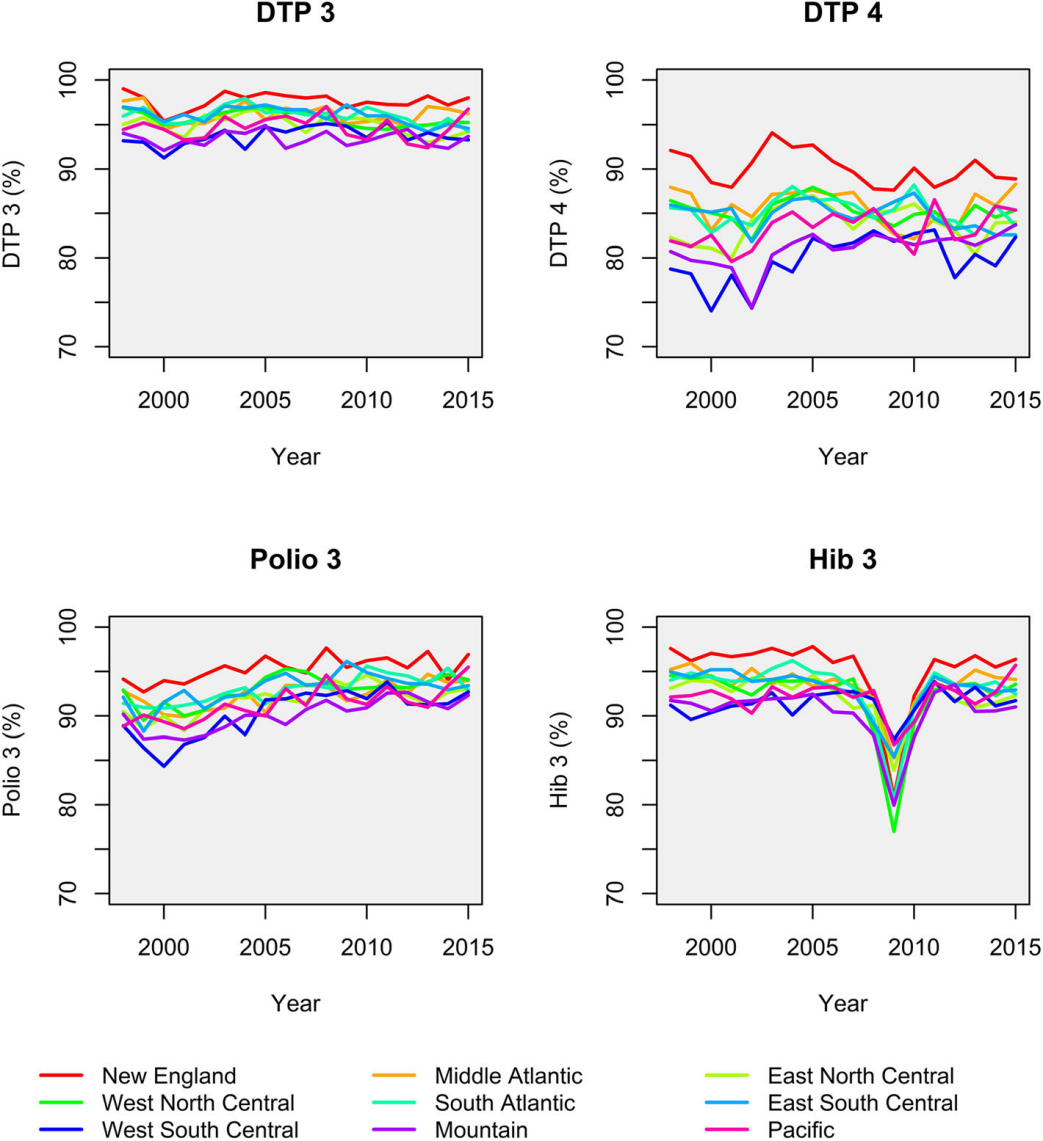
Mean immunization coverage over time, according to U.S. census divisions [14, 26]. Excluding the states Arizona, California, Connecticut, District of Columbia, Idaho, Indiana, Kansas, Kentucky and Nevada from the analyses

The NIS assessed mean immunization coverage in the general population of children aged 19–35 months. Weighted by the number of live births in each state and year, it ranged from 95.3% for 3+ DTP (range 87.9–99.7%), 92.0% for 3+ Polio (range 81.0–98.5%), and 92.4% for 3+ Hib (range 58.8–99.6%) to 83.9% for 4+ DTP (range 66.2–97.0%). Figure 2 shows time trends in immunization coverage according to U.S. divisions.

Table 2 summarizes the association between immunization coverage and SUID in simple and multiple regression analyses. A lower SUID mortality was associated with a higher 4+ DTP coverage by a constant rate ratio (RR) of 0.88 per 10% increase (95% CI = 0.83, 0.95), after adjustment for sleep position and poverty (Table 2, Model 1). When additionally adjusting for race, higher coverage with 3+ DTP (RR 0.76, 95% CI = 0.67, 0.85), 4+ DTP (RR 0.83, 95% CI = 0.78, 0.89), and 3+ Polio (RR 0.81, 95% CI = 0.73, 0.90) was associated with lower SUID mortality (Table 2, Model 2). Coverage with 3+ Hib was not associated with SUID mortality. Table 3 shows the results of simple and multiple regression analyses with SIDS as dependent variable. The results were similar to those of the SUID regression analyses.

**Table 2:** Regression Analysis of Mortality from Sudden Unexpected Infant Death

Dependent variable: deaths from sudden unexpected infant death^a^			
	RR (95% CI)^b^	RR (95% CI)^b^	RR (95% CI)^b^
	Unadjusted	Model 1 -Adjusted for sleep position and poverty	Model 2 -Adjusted for sleep position, poverty and race
		1996–2015	2000–2015
3+ DTP^c^	0.81 (0.72, 0.92)	0.91 (0.80, 1.03)	0.76 (0.67, 0.85)
4+ DTP^d^	0.79 (0.74, 0.84)	0.88 (0.83, 0.95)	0.83 (0.78, 0.89)
3+ Polio^e^	0.92 (0.84, 1.01)	1.00 (0.90, 1.10)	0.81 (0.73, 0.90)
3+ Hib^f^	0.90 (0.84, 0.96)	0.95 (0.89, 1.02)	0.94 (0.88, 1.00)
Supine sleep position	0.90 (0.89, 0.92)	0.94 (0.92, 0.96)	0.95 (0.90, 1.00)
Poverty rate	1.66 (1.55, 1.78)	1.46 (1.35, 1.59)	1.45 (1.31, 1.60)
White American	0.93 (0.91, 0.96)	–	–
Black American	1.23 (1.13, 1.34)	–	–
Asian American	0.47 (0.41, 0.54)	–	–
Race (PC1)^g^	–	–	1.00 (0.97, 1.02)
Race (PC2)^g^	–	–	1.50 (1.42, 1.59)

^a^ Excluding the states Arizona, California, Connecticut, District of Columbia, Idaho, Indiana, Kansas, Kentucky and Nevada from all analyses since no data on sleep position were available (22.3% of all live births)

^b^ Using a simulation (bootstrap) method with *N* = 10.000 runs to determine CI. RR are per 10% increase in the independent variable

^c^ 3 or more doses of any diphtheria and tetanus toxoids and pertussis vaccines, including diphtheria and tetanus toxoids, and any acellular pertussis vaccine (DTP/DTaP/DT)

^d^ 4 or more doses of any diphtheria and tetanus toxoids and pertussis vaccines, including diphtheria and tetanus toxoids, and any acellular pertussis vaccine (DTP/DTaP/DT)

^e^ 3 or more doses of any poliovirus vaccine

^f^ 3 or more doses of *Haemophilus influenzae* type b (Hib) vaccine

^g^ Using the first 2 principal components (PC1 and PC2) for adjustment, with low PC1 values mainly indicating a high percentage of Black and a low percentage of White Americans and low PC2 values indicating a high percentage of Asian Americans

CI indicates confidence interval; DTP, diphtheria-tetanus-pertussis; Hib, *Haemophilus influenzae* type b; PC, principal component; RR, rate ratio

**Table 3:** Regression Analysis of Mortality from Sudden Infant Death Syndrome

Dependent variable: deaths from sudden infant death syndrome^a^			
	RR (95% CI)^b^	RR (95% CI)^b^	RR (95% CI)^b^
	Unadjusted	Model 1 -Adjusted for sleep position and poverty	Model 2 -Adjusted for sleep position, poverty and race
		1996–2015	2000–2015
3+ DTP^c^	0.80 (0.66, 0.96)	0.89 (0.74, 1.08)	0.66 (0.54, 0.81)
4+ DTP^d^	0.78 (0.71, 0.84)	0.85 (0.77, 0.94)	0.74 (0.67, 0.82)
3+ Polio^e^	0.94 (0.81, 1.09)	1.01 (0.87, 1.19)	0.76 (0.65, 0.91)
3+ Hib^f^	0.85 (0.75, 0.96)	0.88 (0.79, 1.00)	0.83 (0.74, 0.92)
Supine sleep position	0.90 (0.87, 0.92)	0.89 (0.87, 0.93)	0.97 (0.90, 1.05)
Poverty rate	1.33 (1.19, 1.49)	1.04 (0.90, 1.20)	1.09 (0.92, 1.29)
White American	0.95 (0.90, 0.99)	–	–
Black American	1.24 (1.04, 1.49)	–	–
Asian American	0.37 (0.29, 0.48)	–	–
Race (PC1)^g^	–	–	0.97 (0.93, 1.02)
Race (PC2)^g^	–	–	1.73 (1.56, 1.92)

^a^ Excluding the states Arizona, California, Connecticut, District of Columbia, Idaho, Indiana, Kansas, Kentucky and Nevada from all analyses since no data on sleep position were available (22.3% of all live births)

^b^ Using a simulation (bootstrap) method with N = 10.000 runs to determine CI. RR are per 10% increase in the independent variable

^c^ 3 or more doses of any diphtheria and tetanus toxoids and pertussis vaccines, including diphtheria and tetanus toxoids, and any acellular pertussis vaccine (DTP/DTaP/DT)

^d^ 4 or more doses of any diphtheria and tetanus toxoids and pertussis vaccines, including diphtheria and tetanus toxoids, and any acellular pertussis vaccine (DTP/DTaP/DT)

^e^ 3 or more doses of any poliovirus vaccine

^f^ 3 or more doses of *Haemophilus influenzae* type b (Hib) vaccine

^g^ Using the first 2 principal components (PC1 and PC2) for adjustment, with low PC1 values mainly indicating a high percentage of Black and a low percentage of White Americans and low PC2 values indicating a high percentage of Asian Americans

CI indicates confidence interval; DTP, diphtheria-tetanus-pertussis; Hib, *Haemophilus influenzae* type b; PC, principal component; RR, rate ratio

We excluded the nine states without any information on sleep position. Results of the unadjusted regression analyses were similar when including all states compared to the main analyses excluding the nine states (Table 4).

**Table 4:** Unadjusted Regression Analyses of Sudden Unexpected Infant Death and Sudden Infant Death Syndrome (all states)

	Sudden unexpected infant death	Sudden infant death syndrome
	RR (95% CI)^a^	RR (95% CI)^a^
3+ DTP^b^	0.89 (0.77, 1.02)	0.88 (0.73, 1.07)
4+ DTP^c^	0.83 (0.78, 0.88)	0.83 (0.76, 0.91)
3+ Polio^d^	0.93 (0.83, 1.04)	1.00 (0.87, 1.15)
3+ Hib^e^	0.91 (0.84, 0.99)	0.88 (0.78, 0.99)

^a^ Using a simulation (bootstrap) method with *N* = 10.000 runs to determine CI. RR are per 10% increase in the independent variable

^b^ 3 or more doses of any diphtheria and tetanus toxoids and pertussis vaccines, including diphtheria and tetanus toxoids, and any acellular pertussis vaccine (DTP/DTaP/DT)

^c^ 4 or more doses of any diphtheria and tetanus toxoids and pertussis vaccines, including diphtheria and tetanus toxoids, and any acellular pertussis vaccine (DTP/DTaP/DT)

^d^ 3 or more doses of any poliovirus vaccine

^e^ 3 or more doses of *Haemophilus influenzae* type b (Hib) vaccine

CI indicates confidence interval; DTP, diphtheria-tetanus-pertussis; Hib, *Haemophilus influenzae* type b; RR, rate ratio

## Discussion

Mortality rates from SUID decreased in the United States between 1992 and 2015. The largest decline occurred between 1992 and 1996, slowing down from 1996 onwards. A slight increase in mortality rates was observed in three out of the nine divisions (New England, East South Central, West South Central) from 1995 onwards. Higher immunization coverage with DTP and polio but not Hib was associated with lower SUID mortality. With regard to SIDS, higher DTP, polio, and Hib immunization coverage was associated with reduced mortality.

A previous study showed an inverse association between SIDS mortality rates and immunization in the United States using historical national data [6]. Data on immunization were, however, based on results of three different surveys over time [28]. In the present study, we used standardized data from the NIS at the state-level, confirming the results of an inverse association between immunization coverage and SUID/SIDS mortality. International comparisons have been hindered by the lack of high-quality data assessing immunization coverage as well as infant sleep position over time. Meta-analyses of case-control and cohort studies have shown a reduced risk of SIDS by immunization, in particular DTP and polio [11, 12]. While case-control and cohort studies allow the determination of risk on the individual level, ecological studies yield information on the association with immunization coverage at the population level.

Immunization may protect infants from SUID and SIDS in various ways. An infectious cause for SUID and SIDS cannot be excluded. A mild tracheobronchial inflammation has been observed in about half of SIDS cases [10]. Immunization may provide direct and indirect protection against specific agents such as *Bordetella pertussis* [6, 29]. Other potential pathways include the prevention of infections by other agents and/or the reduction of vulnerability in infants following infections. Further research into these hypotheses is needed.

Immunization coverage varied by type of vaccine and over time. While the 3+ doses of DTP, polio, and Hib achieved a mean coverage of at least 90%, only 84% of the children received 4+ doses of DTP. The regional variation in the uptake of the 4+ DTP was greater compared to the 3+ DTP, 3+ polio, and 3+ Hib. Shortages in vaccine supply were reported for both pertussis and Hib [30, 31]. The shortage in the pertussis vaccine in the United States from 2000 to mid-2003 affected particularly infants in public clinics and in the Southern Census region [30]. The NIS may underestimate the shortage, as only approximately 15% of children in the NIS were from public clinics (2001–2002). One measure to counteract the shortage was the deferral of the 4th dose of DTP. With regard to the decrease in Hib immunization coverage in 2009, a shortage of the Hib vaccine was reported - starting in December 2007 - due to the recall of several lots of the vaccines PedvaxHIB® and Comvax® by the manufacturer [31].

A major limitation of the comparison of time trends are changes in diagnostic coding [4]. A diagnostic shift from SIDS to other diagnoses may have occurred as recommendations and standard protocols for death scene investigation have been developed further [32, 33]. Regional variations may exist in the likelihood of death certifiers classifying infant deaths as SIDS [4, 34]. Using SUID as diagnostic group allows for the comparison of mortality rates over time and across regions, taking into account changes in diagnostic practices. Another limitation is that we only included data on immunization coverage at the state-level. With regard to immunization, larger disparities may exist on the local level [30]. Finally, we had to impute data on sleep position at the state-level for a number of years and states. We had to exclude nine out of the 50 states and the District of Columbia with completely missing data on sleep position. For most of the 42 remaining states, only incomplete information on sleep position was available. We treated missing data using multiple imputation with m = 5 samples. Since the reasons for missing data on sleep position were unrelated to sleep position, poverty, race, and SUID/SIDS rates we assumed that missing data was completely at random. Under this assumption, multiple imputation is not a source of bias.

## Conclusions

SUID mortality decreased in the United States; however, the decline has slowed down since 1996. We conclude that immunization coverage is inversely related to SUID mortality. Although the overall immunization coverage is high, large variations exist between states. The protective effect of immunization may be due to the prevention of specific infections and/or to a reduced vulnerability in infants because of an overall lower rate of infections. The potential role of infections in the etiology of SUID requires further research. Independent from the investigation of pathophysiologic mechanisms, public health efforts are needed to reduce regional variations in immunization coverage. Achieving high immunization coverage will protect infants not only from specific infections but potentially from SUID as well.

## Data Availability

The datasets analysed during the current study are publicly archived datasets and available using the hyperlinks as referenced. All code to download and replicate the publicly available data, as well as to reproduce all tables and plots, will be uploaded at https://github.com/.

## Abbreviations

CDC: Centers for Disease Control and Prevention
CI: Confidence interval
DTP: Diphtheria-tetanus-pertussis
DTaP: Diphtheria-tetanus-acellular pertussis
Hib: *Haemophilus influenzae* type b
ICD: International Classification of Deaths
NIS: National Immunization Survey
PC: Principal components
PRAMS: Pregnancy Risk Assessment Monitoring System
SIDS: Sudden infant death syndrome
SUID: Sudden unexpected infant death
WONDER: Wide-ranging Online Data for Epidemiologic Research
